# Electronic health record-based assessment of oral corticosteroid use in a population of primary care patients with asthma: an observational study

**DOI:** 10.1186/1710-1492-9-27

**Published:** 2013-08-07

**Authors:** Felicia C Allen-Ramey, Linda M Nelsen, Joseph B Leader, Dione Mercer, Henry Lester Kirchner, James B Jones

**Affiliations:** 1 Merck & Co. Inc., West Point, PA 19486, USA; 2 Geisinger Clinic, Center for Health Research, Danville, PA 17822, USA; 3 Geisinger Clinic, Division of Medicine, Danville, PA 17822, USA

**Keywords:** Oral corticosteroids, Asthma, Anti-asthmatic agents, Retrospective studies, Therapeutic use, Managed care programs, Cross-sectional studies

## Abstract

**Background:**

Oral corticosteroid prescriptions are often used in clinical studies as an indicator of asthma exacerbations. However, there is rarely the ability to link a prescription to its associated diagnosis. The objective of this study was to characterize patterns of oral corticosteroid prescription orders for asthma patients using an electronic health record database, which links each prescription order to the diagnosis assigned at the time the order was placed.

**Methods:**

This was a retrospective cohort study of the electronic health records of asthma patients enrolled in the Geisinger Health System from January 1, 2001 to August 23, 2010. Eligible patients were 12–85 years old, had a primary care physician in the Geisinger Health System, and had asthma. Each oral corticosteroid order was classified as being prescribed for an asthma-related or non-asthma-related condition based on the associated diagnosis. Asthma-related oral corticosteroid use was classified as either chronic or acute. In patient-level analyses, we determined the number of asthma patients with asthma-related and non-asthma-related prescription orders and the number of patients with acute versus chronic use. Prescription-level analyses ascertained the percentages of oral corticosteroid prescription orders that were for asthma-related and non-asthma-related conditions.

**Results:**

Among the 21,199 asthma patients identified in the electronic health record database, 15,017 (70.8%) had an oral corticosteroid prescription order. Many patients (N = 6,827; 45.5%) had prescription orders for both asthma-related and non-asthma-related conditions, but some had prescription orders exclusively for asthma-related (N = 3,450; 23.0%) or non-asthma-related conditions (N = 4,740; 31.6%). Among the patients receiving a prescription order, most (87.5%) could be classified as acute users. A total of 60,355 oral corticosteroid prescription orders were placed for the asthma patients in this study—31,397 (52.0%) for non-asthma-related conditions, 24,487 (40.6%) for asthma-related conditions, and 4,471 (7.4%) for both asthma-related and non-asthma-related conditions.

**Conclusions:**

Oral corticosteroid prescriptions for asthma patients are frequently ordered for conditions unrelated to asthma. A prescription for oral corticosteroids may be an unreliable marker of asthma exacerbations in retrospective studies utilizing administrative claims data. Investigators should consider co-morbid conditions for which oral corticosteroid use may also be indicated and/or different criteria for assessing oral corticosteroid use for asthma.

## Background

The prevention of exacerbations of asthma symptoms is recognized in treatment guidelines as a key component of controlling asthma [1]. An exacerbation is broadly defined as an acute worsening of asthma symptoms requiring a transient change in treatment. There is, however, neither a uniformly accepted definition of an exacerbation nor a consensus on how an exacerbation should be measured [2].

The American Thoracic Society and European Respiratory Society (ATS/ERS) recommended in an official statement that the definition of a severe asthma exacerbation should include the use of systemic corticosteroids [2]. However, this definition applies only in clinical trials [2], and subjects enrolled in major randomized clinical trials of asthma medications are not representative of asthma patients in the general population [3]. Asthma patients in the general population are often studied retrospectively by way of administrative data sets in which surrogate variables are used as a measure of exacerbations.

Observational studies of administrative data sets have used oral corticosteroid prescriptions, either alone or as part of a composite measure with asthma-related emergency department visits and inpatient stays, as a measure of asthma exacerbations [4-10]. The definitions of an exacerbation in these types of studies vary extensively. Finkelstein et al. used the “dispensing of an oral steroid preparation as a proxy for the occurrence of an acute exacerbation” [11]. Friedman defined an asthma exacerbation as an episode requiring “hospitalization, treatment in an emergency room, or an outpatient visit” where the patient “received nebulized medication or a prescription for oral corticosteroids” [12]. More recent studies have been more explicit in terms of days’ supply of oral corticosteroid and the timing of a prescription relative to an outpatient visit [7,13,14]. However, the lack of a standardized definition of exacerbations limits the applicability of observational findings to clinical practice.

The NIH and the Agency for Healthcare Research and Quality have drafted recommendations for the standardization of asthma outcome measures for both clinical trials and observational studies [15]. The recommendations define an exacerbation as a “worsening of asthma requiring the use of systemic corticosteroids to prevent a serious outcome” [16]. For exacerbations in patients twelve years of age and older, the core outcome measures for observational studies are the use of systemic corticosteroids (oral, injected, or IV), hospitalization, and emergency department (or urgent care) visits for asthma [16].

One limitation of using oral corticosteroids as a measure of asthma exacerbations is that administrative claims data sets typically do not link a claim for a prescription to the diagnosis prompting the prescription, making it difficult to determine whether a given oral corticosteroid prescription was written for an asthma exacerbation or for a condition unrelated to the patient’s asthma. Oral corticosteroids can be prescribed for variety of conditions unrelated to asthma, including systemic inflammatory diseases and pain syndromes. Integrated health care delivery systems that maintain electronic health records of all patient encounters contain the information needed to link a prescription order to the diagnosis assigned at the time the order was placed. Thus, with an electronic health record database, it is possible to determine whether an oral corticosteroid prescription was ordered for an asthma exacerbation or for some other reason.

The objective of this study was to use the electronic health records of an integrated health care delivery system to determine the medical diagnoses associated with oral corticosteroid prescriptions ordered for asthma patients, and thus to characterize the use of oral corticosteroid prescriptions as a measure of asthma exacerbations in a primary care setting.

## Methods

### Study design and setting

This study was a retrospective cohort analysis of oral corticosteroid use in a population of primary care asthma patients who are members of the Geisinger Health System, an integrated health care delivery system that serves residents in central and northeastern Pennsylvania. The Geisinger Health System includes the Geisinger Clinic, a network of 37 community-based offices staffed by primary care physicians. The Geisinger Clinic network provides primary care to over 400,000 patients. All network offices have used the EpicCare™ electronic health record (EHR) since 2001. The study period was January 1, 2001 to August 23, 2010.

### Data sources

Geisinger’s longitudinal EHR served as the data source for this study. The EHR includes a patient’s “problem list”, a dynamic and comprehensive list of all of a patient’s medical problems. Each medical problem is entered into the EHR by a provider (e.g., nurse or physician) using a structured vocabulary, the terms of which are automatically linked to the International Classification of Disease (ICD-9) code. Similarly, all patient encounters (e.g., office visits) are assigned at least one diagnostic ICD-9 code by the provider. These provider-entered “encounter diagnosis codes” summarize the specific medical problems addressed during the encounter and are used also for billing purposes. Whereas the problem list is a comprehensive, current list of all of a patient’s medical problems and can change over time as problems are resolved and/or new problems are identified, encounter diagnoses are encounter-specific—once assigned to an encounter, they do not change. The EHR includes the date that each problem is added to or removed from the problem list.

At all Geisinger Clinic offices, medication prescriptions must be ordered electronically via the EHR. Each order includes the following items: order date; medication name, dose, and class; prescribed quantity; allowed number of refills; and free-text instructions on dosing and administration (the medication “sig”). In addition, the provider is required to assign one or more diagnostic codes that summarize the conditions the medication is intended to treat. As with the problem list and encounter codes, the reason for the prescription order is entered using a structured vocabulary and mapped to an underlying ICD-9 code.

### Patient sample

Potentially eligible patients were identified by searching Geisinger’s EHR. The sampling frame included all patients aged 12 to 85 with a primary care provider at one of the Geisinger clinics and an indication of having asthma during the study period. Patients were classified as having asthma if they had any one of the following: ≥2 office visits within a 12-month period with an associated ICD-9 code of 493.xx, an active asthma diagnosis on their problem list during the study period, or a prescription order with an associated asthma diagnosis (493.xx).

Patients were excluded if they had ICD-9 codes for chronic obstructive pulmonary disease (491.xx, 492.xx), bronchiectasis (494.xx), chronic airway obstruction (not otherwise specified; 406.xx), cystic fibrosis (748.4), or bronchopulmonary dysplasia (770.7) on their problem list or in an encounter diagnosis. The study was approved with a waiver of patient consent by the Geisinger Health System institutional review board (Ref: 2010-0297).

### Oral corticosteroid prescriptions analysis

After identifying all eligible asthma patients, we identified every oral corticosteroid prescription ordered for an asthma patient during the study period. Each order was classified on two dimensions: relation to asthma (yes or no) and pattern (acute or chronic). To determine whether an oral corticosteroid order was asthma-related, we relied on the diagnosis code assigned to the order by the provider. In collaboration with clinical experts, we classified ICD-9 codes 493.00 (extrinsic asthma, unspecified), 493.02 (extrinsic asthma with exacerbation), 493.90 (unspecified asthma), and 493.92 (unspecified asthma, with exacerbation) as asthma-related; all other codes were classified as non-asthma-related.

Whether the pattern of oral corticosteroid orders was acute or chronic was determined on the basis of the order information in the EHR (the details of which were described above). The definitions of acute and chronic use of oral corticosteroids were derived after investigator review (FAR and LN) of a random sample of prescription orders. The initial definition was then applied to a subsequent sample of orders for validation. The final definition used for the analysis (described below) was endorsed through a third sample of orders reviewed alongside the proposed definition by a pulmonary physician with the Geisinger Health System. An acute order was defined as (1) an order with zero refills or (2) an order quantity <30. A chronic order was defined as an order quantity ≥30 with one or more refills. These resulting criteria were then applied to all oral corticosteroid orders for patients in the study population.

### Data analysis

Because a single patient can have multiple prescription orders, separate descriptive analyses were conducted using, first, the patient, and second, the prescription order, as the unit of analysis. Patient-level analyses included: the number of patients with asthma, the number of asthma patients with asthma-related and non-asthma-related oral corticosteroid orders, and the number of asthma patients with patterns of oral corticosteroid orders consistent with acute versus chronic use. Prescription-level analyses included the total number of oral corticosteroid orders for asthma patients and the percentages of those orders written for asthma-related versus non-asthma-related conditions. Differences in acute and chronic order patterns along with diagnoses associated with OCS orders were examined for adolescents (ages 12–17 years), adults (18–44 and 45–64 years) and elderly patients (≥65 years).

## Results

### Characteristics of the asthma patient population

**Figure 1:**
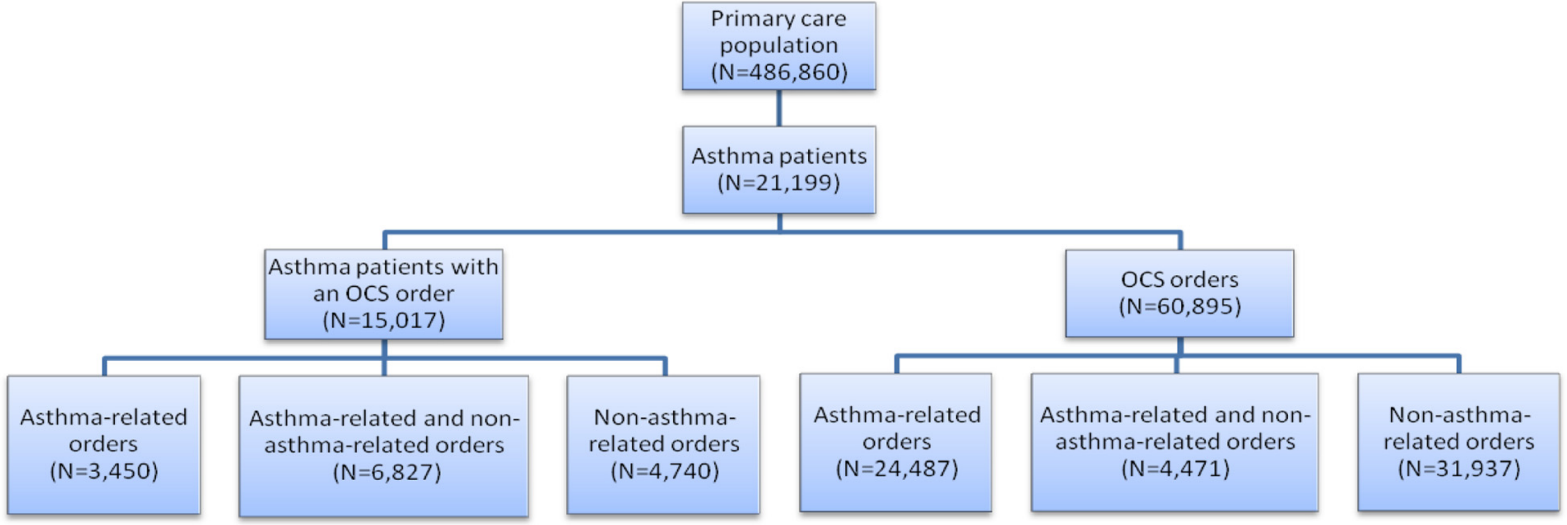
**Number and type of oral corticosteroid prescription orders for asthma patients.** OCS, oral corticosteroid.

A total of 21,199 unique asthma patients were identified in the EHR database for the study period and met the study inclusion criteria (Figure 1; Table 1). The mean age was 39.5 years, and majorities of the patients were female (67.9%) and Caucasian (94.3%). The total average number of office visits per patient was 36.5, while the average number of asthma-related office visits was 8.3. Similarly, the average number of asthma-related emergency department visits and inpatient visits (1.6 and 1.5, respectively) was only a fraction of the total number of each type of visit (3.6 and 3.5, respectively). The most prevalent comorbidities, present in about half the study population, were acute sinusitis (58.6%), allergic rhinitis (56.1%), and acute bronchitis (48.8%).

**Table 1:** **Characteristics of the asthma patient population**^**a**^

	**Age 12–17**		**Age 18–64**		**Age ≥ 65**		**Total**	
	**N = 3,496**		**N = 15,352**		**N = 2,351**		**N = 21,199**	
Age, years (mean, SD)	14.3	1.7	40.1	12.4	72.8	5.8	39.5	18.5
Female	1,879	54%	10,859	71%	1,650	70%	14,394	67.9%
Caucasian	3,181	91%	14,500	94%	2,304	98%	19,990	94.3%
Encounters, per patient (mean, SD)^b^								
Total office visits	27.6	23.1	36.6	31.7	48.6	35.4	36.5	31.4
Asthma-related office visits	6.3	5.4	8.5	8.0	10.3	9.8	8.3	7.9
Total ED visits	3.5	5.1	3.8	7.9	2.8	4.5	3.6	7.3
Asthma-related ED visits	1.7	1.1	1.5	1.4	1.4	1.1	1.6	1.6
Total inpatient visits	2.7	4.1	3.1	4.4	5.1	7.1	3.5	5.1
Asthma-related inpatient visits	1.5	1.3	1.5	1.4	1.4	1.1	1.5	1.4
Comorbidities								
Acute sinusitis	2,129	61%	9,353	61%	938	40%	12,420	58.6%
Allergic rhinitis	2,193	63%	8,733	57%	957	41%	11,883	56.1%
Acute bronchitis	1,167	33%	7,935	52%	1,248	53%	10,350	48.8%
Allergic rhinorrhea	1,719	49%	6,580	43%	666	28%	8,965	42.3%
Acute upper respiratory conditions	1,772	51%	5,700	37%	662	28%	8,134	38.4%
Gastroesophageal reflux disease	764	22%	6,159	40%	1,142	49%	8,065	38.0%
Chronic sinusitis	376	11%	2,458	16%	297	13%	3,131	14.8%
Chronic otitis media	755	22%	1,977	13%	131	6%	2,863	13.5%
Pneumonia	312	9%	1,523	10%	479	20%	2,314	10.9%
Current Smoker	515	15%	2,234	15%	52	2%	2,801	13.2%

*ED* emergency department, *SD* standard deviation.

^a^Data are presented as N (%) unless otherwise indicated.

^b^Office visits were calculated over the period 2001–2010, ED visits and inpatient visits were calculated over 2004–2010.

### Asthma patients with oral corticosteroid prescription orders

Of the 21,199 asthma patients, 15,017 (70.8%) had a prescription order for an oral corticosteroid. Among all patients with an oral corticosteroid prescription order, 3,450 (23.0%) had orders exclusively for an asthma-related condition, 4,740 (31.6%) had orders exclusively for a non-asthma-related condition, and 6,827 (45.5%) had orders for both asthma-related and non-asthma-related conditions. Most asthma patients (87.5%) were classified as acute users regardless of age (Table 2).

**Table 2:** Number of asthma patients with oral corticosteroid order patterns classified as acute or chronic

	**Age 12–17**	**Age 18–44**	**Age 45–64**	**Age ≥ 65**	**Total**
	**(N = 1,995)**	**(N = 6,891)**	**(N = 4,464)**	**(N = 1,667)**	**(N = 15,017)**
Acute	1,919 (96.2%)	6,249 (90.7%)	3,703 (83%)	1,263 (75.8%)	13,134 (87.5%)
Chronic	76 (3.8%)	642 (9.3%)	761 (17%)	404 (24.2%)	1,883 (12.5%)

### Oral corticosteroid prescription orders for asthma patients

A total of 60,355 oral corticosteroid prescription orders were placed for the asthma patients in this study—24,487 (40.6%) for an asthma-related condition, 31,397 (52.0%) for non-asthma-related conditions, and 4,471 (7.4%) for both asthma-related and non-asthma-related conditions (Figure 1). Among patients with one or more prescriptions for oral corticosteroids, the median number (range) of oral corticosteroid prescription orders per patient was 2.0 (1–103) for asthma-related conditions and 2.0 (1–72) for conditions unrelated to asthma. The most common diagnoses associated with oral corticosteroid prescription orders among patients with asthma are listed in Table 3.

**Table 3:** Diagnoses associated with oral corticosteroid orders for patients with asthma

**Diagnosis category**	**Age 12-17**		**Age 18-44**		**Age 45-64**		**Age ≥ 65**		**Total**	
	**Count**^**a**^	**Percent**^**b**^	**Count**^**a**^	**Percent**^**b**^	**Count**^**a**^	**Percent**^**b**^	**Count**^**a**^	**Percent**^**b**^	**Count**^**a**^	**Percent**^**b**^
Asthma-related	3185	4.2%	13463	17.9%	9356	12.4%	2954	3.9%	28958	38.5%
Other^c^	661	0.9%	3987	5.3%	4251	5.6%	1509	2.0%	10408	13.8%
Other upper respiratory^d^	1008	1.3%	4522	6.0%	3130	4.2%	795	1.1%	9455	12.6%
Pain syndrome	316	0.4%	3505	4.7%	3196	4.2%	1100	1.5%	8117	10.8%
Missing	368	0.5%	2168	2.9%	1635	2.2%	867	1.2%	5038	6.7%
Allergic reaction	682	0.9%	2043	2.7%	1258	1.7%	364	0.5%	4347	5.8%
Systematic inflammatory	115	0.2%	1028	1.4%	1670	2.2%	1264	1.7%	4077	5.4%
Other lower respiratory	255	0.3%	775	1.0%	611	0.8%	271	0.4%	1912	2.5%
Other skin	177	0.2%	695	0.9%	453	0.6%	157	0.2%	1482	2.0%
Neoplasms	34	0.0%	194	0.3%	350	0.5%	220	0.3%	798	1.1%
Infection	18	0.0%	223	0.3%	277	0.4%	39	0.1%	557	0.7%
Unsure	11	0.0%	44	0.1%	34	0.0%	20	0.0%	109	0.1%
COPD	1	0.0%	6	0.0%	13	0.0%	14	0.0%	34	0.0%

^a^Column total does not equal the total number of distinct orders because each individual order can potentially have multiple diagnosis categories. Each diagnosis category represents a distinct count of orders.

^b^Percentages were calculated relative to the total number of prescription diagnoses (N = 75,292) associated with all OCS medication orders (N = 60,355) among asthma patients.

^c^Other includes included pain syndrome, endocrine, head and neck disorders, hematologic, infections and autoimmune conditions.

^d^Other upper respiratory includes rhinitis (allergic and chronic), chronic sinusitis, nasal polyps, tonsillitis and other conditions of the septum and larynx.

## Discussion

In this population of primary care patients, over half (61.5%) of the oral corticosteroid prescriptions written for patients with asthma were prescribed for conditions unrelated to asthma (Table 3). This result suggests that, without an associated diagnosis, a prescription for an oral corticosteroid is not by itself an adequate marker for an asthma exacerbation in patients with asthma (but without other chronic respiratory diseases). This is consistent with evidence that a systemic corticosteroid prescription is poorly predictive of a diagnosis of asthma [17].

Oral corticosteroid use meets three of the four requirements for a core outcome measure in the NIH draft recommendations: it is clinically relevant, feasible, and enables comparison across studies [15]. The fourth requirement, evidence of validity, requires further research. In particular, the factors that contribute to patient and clinician decisions to use oral corticosteroids need to be investigated [16]. This study is, to our knowledge, the first to report the frequency with which asthma patients receive oral corticosteroids for conditions unrelated to asthma.

Across all age groups in this sample, most asthma patients were acute users of oral corticosteroids. A minority (12.5% overall) were considered chronic users. However, frequent exacerbations are difficult to distinguish from poorly controlled persistent asthma. The chronic asthma-related use by some patients—the number of prescriptions ranged up to 103, i.e., 8 years of 30-day prescriptions—may represent poor control of persistent asthma due to inadequate use of long-term control medications (inhaled corticosteroids, etc.) rather than exacerbations. (‘Chronic’ use of systemic corticosteroids is an option to control severe asthma [1]). Thus, only the acute use (which was 87.5% of all use) can reliably be related to exacerbations.

The principal limitation of this analysis is uncertainty about the diagnosis associated with prescription orders. Physicians are required to enter a diagnosis code on each prescription order, but there is no quality check to ensure that the selected code is accurate. As a result, misclassification of asthma-related versus non-asthma-related prescription orders might occur. A small number (9.4%) of the oral corticosteroid orders in the EHR did not have an associated diagnosis, and these were categorized as being non-asthma-related in our analysis. Given the potential for orders classified as upper respiratory but not asthma-related to alter our results, we conducted a sensitivity analysis classifying bronchitis as an asthma-related condition. The resulting proportion of oral corticosteroid orders that were not asthma-related declined from 61.5% to 55.0%, which still supports our conclusions. In addition, the appearance of ‘asthma’ in a patient’s problem list in an EHR has not been validated as a means of diagnosing asthma. Hence, it is possible that patients identified as having asthma solely on the basis of an entry in the patient’s problem list did not actually have asthma. The validity of the use of health administrative databases to identify asthma patients, however, has been studied. In this study, patients were classified as having asthma if they had ≥2 office visits within a 12-month period with an associated ICD-9 code for asthma. Gershon et al. (2009) reported that a similar algorithm (≥2 ambulatory care visits and/or ≥1 hospitalization for asthma in a 2 year period) had a sensitivity and specificity of 83.8% and 76.5%, respectively, with expert chart review as the reference standard [18]. The positive predictive value was 61.5% (but 72.5% with the primary care physician chart diagnosis as reference standard) [18].

This analysis did not account for the chronology of the oral corticosteroid prescription with respect to exacerbations. Unless oral corticosteroid orders were recorded only *after* a diagnosis of asthma was entered into the EHR, it is possible that patients did not have asthma when the oral corticosteroid was prescribed. One way to improve this study design is to define the chronology of the prescription with respect to the asthma-related outpatient visit, as was done by Schatz et al., who noted that some oral corticosteroids may be prescribed prophylactically rather than for a current exacerbation [7]. Another way is to impose a limit on the time between an outpatient visit for asthma and a prescription order related to asthma (e.g., ≤5 days), as was done by Lee et al. [19]. A second level of stringency could include defining the days’ supply of oral corticosteroid, as was done in two recent studies that defined exacerbations by oral corticosteroid prescriptions for <22 days’ supply [13,14]. These approaches combined would ensure that the prescription was truly for an acute exacerbation and not the result of inadequate control of persistent asthma symptoms.

## Conclusions

In this population of primary care patients, over half of the oral corticosteroid prescriptions for patients with asthma were for conditions unrelated to asthma. This finding has implications for how oral corticosteroid prescriptions should be interpreted in future observational research, particularly retrospective studies. In studies utilizing administrative claims data, a prescription for oral corticosteroids may be an unreliable marker of asthma exacerbations. Investigators should consider co-morbid conditions for which oral corticosteroid use may also be indicated. The accuracy of an oral corticosteroid prescription as a marker of an exacerbation should be evaluated, and algorithms to identify oral corticosteroid prescriptions ordered for acute asthma episodes in administrative claims data should be developed and validated.

## Abbreviations

ATS/ERS: American thoracic society and european respiratory society; EHR: Electronic health record; ICD: International classification of disease.

## Competing interests

FAR and LN are employees of Merck & Co., Inc., which funded the study. JL, DM, HLK, and JBJ are employees of the Geisinger Clinic, whose electronic health record database was the source of the data for this study.

## Authors’ contributions

FAR and LN in conjunction with JBJ and HLK conceived of the study and participated in its design while DGM provided input and coordination. JL performed the statistical analysis. All authors participated in drafting the manuscript and have read and approved the final version.
